# Evaluation of a single-use bioartificial liver (BAL) biocartridge consisting of cryopreservable alginate encapsulated liver cell spheroids as a component of HepatiCan™, a novel bioartificial liver device

**DOI:** 10.3389/fbioe.2025.1572254

**Published:** 2025-08-01

**Authors:** Eloy Erro, Tom Brookshaw, Barry Fuller, Sweta Chandel, Joana Mendonca da Silva, Elizaveta Zotova, Sherri-Ann Chalmers, Alfie Watt, Clare Selden

**Affiliations:** ^1^ UCL Institute for Liver and Digestive Health, UCL Medical School, London, United Kingdom; ^2^ UCL Division of Surgery and Interventional Science, UCL Medical School, London, United Kingdom

**Keywords:** bioartificial liver (BAL), single-use biocartridge, computational fluid dynamics (CFD), alginate encapsulated liver spheroids (AELS), cryopreservation, hydrodynamic conditions, metabolic activity

## Abstract

**Introduction:**

alternative therapies to complement liver transplantation and treat patients with liver failure are not available. In this study, a clinical scale single-use biocartridge was developed for use as part of a novel Bioartificial Liver device (HepatiCan™), utilising conditioned human-derived alginate encapsulated liver spheroids (AELS), within a fluidised bed.

**Methods:**

to develop the optimal biocartridge, two designs (B2 and B3) were created and modelled to best replicate the performance of our preexisting reusable cartridge (B1). The suitability of designs, and their ability to deliver the required hydrodynamic conditions for AELS, during both spheroid production and treatment phases, was addressed by computational fluid dynamics (CFD). Subsequently, the B3 biocartridge was produced and tested under continuous fluidisation conditions for the growth after encapsulation and recovery after cryopreservation of micro-spheroids in hydrogel scaffolds (AELS).

**Results:**

the main difference between the designs in (B2 and B3) was the base plate flow distributor. Preserving the hole pattern in the base plate, between B1 and B3, was critical for mimicking fluid flow. Additionally, increasing the number of orifices in the cross-patterned base plate design (B3) provided further benefits: maintaining homogeneity in fluid velocity distribution, whilst avoiding “dead-flow” zones. During AELS culture (using B3 format), a cell density of 24.27 ± 3.0 × 106 cells/mL of beads was achieved by day 11. Additionally, post-thaw recovery (PTR) culture of previously cryopreserved clinical doses of AELS was performed for up to 4 days. Return to the pre-freeze total biomass (6.34 ± 0.9 × 1010 cells of AELS) was achieved after 3 days of PTR; AELS growth continued to a total biomass of 8.48 ± 1.6 × 1010 cells by 4 days.

**Discussion:**

the final biocartridge design (B3) was as effective in fluid distribution as the original (B1). B3 surpassed B1 in velocity uniformity over the first 10 mm above the base plate, critical for good mass transfer between biomass and perfusing fluid in the fluidised bed. Sustained biological function for AELS after PTR was demonstrated. One remarkable advantage of this biocartridge is the recovery of functional AELS biomass after cryopreservation. Thus, we facilitate the off-the-shelf availability, whilst preserving essential biological functionality.

## 1 Introduction

Acute liver failure (ALF) is the rapid loss of liver function, which can progress quickly and irreversibly to severe life-threatening complications. Some of the most common symptoms of ALF include jaundice, mental confusion or hepatic encephalopathy, bleeding due to impaired blood clotting, and swelling in the legs and ankles. In severe cases, ALF leads to coma and eventually death (Ostapowicz et al., 2002). To date the only effective treatment for ALF is liver transplantation (Ala-Kokko et al., 1987; Riordan and Williams, 2008), as a full repertoire of liver function is required to support physiology and enable survival. Unfortunately, there is a shortage of livers available for transplant (Arulraj and Neuberger, 2011; Dutkowski et al., 2011), compounding the problems of treating ALF.

Several approaches have been developed for liver failure treatment, both artificial support and bioartificial livers (Carpentier et al., 2009). Artificial liver support devices lack a cellular component and are based on alternative methods for patient treatment, such as detoxification using chemical adsorption for toxin removal together with, for example, albumin dialysis or ion exchange. No artificial liver devices have improved patient survival to date (Kjaergard et al., 2003; O'Grady et al., 1988; Riordan and Williams, 2008), suggesting a more complex and holistic support is required. Due to the complex bio-functionality of the liver, a purely detoxification-based method is unlikely to be sufficient. Moreover, the synthetic function of the liver is also necessary to reproduce the full repertoire of liver functions, i.e., synthetic, metabolic and detoxification (Gumucio et al., 1988).

Selecting the appropriate biomass for BAL treatment is crucial. Several therapies utilise porcine hepatocytes (Demetriou et al., 2004; Mazariegos et al., 2001; Sauer et al., 2003; Van De Kerkhove et al., 2002), which are more readily available. However, porcine cells pose a risk of zoonotic disease transmission (Fruhauf et al., 2009), including endogenous retrovirus xeno-transmission. Whilst there is a lack of availability of primary human hepatocytes in sufficiently large numbers as a cell source for a BAL, the use of well-differentiated liver cell lines becomes an attractive alternative.

We addressed the urgent need for an effective bioartificial liver, by developing HepatiCan™, a Bioartificial Liver (previously named UCLBAL) (Opie et al., 2024). The technology utilises multicellular spheroids of a GMP-rederived human liver cell line (HepG2) encapsulated in alginate in a biocartridge, with fluidisation of the microbeads (Erro et al., 2013; Selden et al., 2017; Selden et al., 2013). The concept of a fluidised bed bioreactor based on alginate encapsulated liver spheroids (AELS), is the resuspension of a solid phase, alginate cell beads, in a liquid phase (e.g., culture media or plasma), homogeneously creating an expanded bed of micro-beads (Doré and Legallais, 1999; Legallais et al., 2000; Naghib et al., 2017). These micro-beads experience both an upward force from the fluid flow from below, and a downward gravitational movement, creating a continuous circulating loop. An expanded AELS bed enables optimal diffusion of nutrients or removal of toxins. This 3-dimensional microgravity environment is advantageous for the treatment of patient plasma, due to high mass transfer between homogeneously dispersed cell spheroids and plasma. As a result, a high mass transfer coefficient and low hydrodynamic shear stresses are generated (David et al., 2004; Gautier et al., 2011), providing prolonged BAL support for failing livers.

Another advantage of the use of AELS as a micro-liver biomass unit, is the flexibility it offers, with uniform size (approximately 0.5 mm in diameter) of the alginate micro-beads. Beads offer control of gravitational density by physical means, with a density modifier. Cryopreservation of AELS has also been achieved in both small volume containers and cryobags containing volumes suitable for clinical scale use (Kilbride et al., 2016; Kilbride et al., 2017; Kilbride et al., 2014; Massie et al., 2011; Puschmann et al., 2017). Both the size of encapsulated beads (∼500 µm) and the multiple spheroids within, provide advantages for cryopreservation: uniform penetration of the cryoprotectants, fewer temperature gradients during freezing and thawing, and minimal mechanical stress during the freezing and thawing processes (Gurruchaga et al., 2018; Matsumoto et al., 2001; Zhang et al., 2018). To compare flow dynamics (fluidisation studies and mixing efficiency) we produced empty alginate beads (EAB) of the same dimensions, using the same encapsulation process, reducing cost and experimental time.

In this study we tested the hypothesis that a single-use disposable cartridge can be designed, manufactured and tested, utilising theoretical mathematical modelling of fluid flows as the initial frame of reference. This reduces the time and cost of producing a new medical device suitable for use in patients to deliver a novel cell therapy. Supported by computational fluid dynamic modelling, a reusable biocartridge was reverse-engineered and manufactured in medical-grade materials as one component of a combined Advanced Therapy Medicinal Product (ATMP), HepatiCan™. Fluid modelling was used to select the optimum biocartridge design, which was then tested for suitability in two use-stages: (i) initial AELS formation and conditioning after cell encapsulation; and (ii) recovery and potency assessment over several days after cryopreservation. The synthetic function of the biomass was assessed during Fluidised Bed Bioreactor (FBB) culture, measured as “per cell performance,” both before and during recovery from cryopreservation. The system has been developed to handle cell doses up to the litre scale, and sustain AELS recovered from cryopreservation.

## 2 Materials and methods

### 2.1 Physical characteristics of the reusable biocartridge (B1)

The reusable biocartridge (B1) was designed to create expanded bead bed conditions, for creating microgravity and favouring alginate encapsulated liver spheroid (AELS) formation. The concept has been extensively tested in both proliferation and treatment phases during two pre-clinical trials (Selden et al., 2017; Selden et al., 2013).

Design details included a fluid inlet positioned centrally at the bottom which acted as a narrow vertical inflow channel which divided into two functional components. A horizontal flow splitter, with four outlets orientated at 90° between them, directing the fluid against the biocartridge walls for mixing. The area between the flow splitter and the base plate allowed additional fluid mixing. The perforated base plate acted as a second flow distributor directing the fluid upwards. Two central and interconnected silicone tubing coils, made from gas-permeable USP Class VI material, facilitated oxygen transfer to the inner culture medium during AELS growth and recovery after cryopreservation. A sealed hollow metal rod at the top of the biocartridge was used for temperature monitoring, containing a temperature probe (PT100). A stainless steel 300 µm cylindrical mesh filter at the top and a 200 µm stainless steel sheet mesh above the base plate, prevented alginate beads from escaping the biocartridge. The whole unit was contained within a glass cartridge (suitable only for experimental work), but which could be disassembled and sterilised for re-use (Figaro et al., 2015; Van De Kerkhove et al., 2002; van der Hilst et al., 2009).

### 2.2 3D biocartridge drawings and fluid flow modelling simulation

Two alternative designs (B2 and B3) were modelled and compared with B1, built previous to these studies using materials that did not meet medical regulations. The new design should replicate or improve the fluid dynamics of the reusable biocartridge (B1). The suitability of the new designs was then tested, theoretically, using computational fluid dynamics (CFD).

All biocartridge models were created using SolidWorks^®^ v.2018, a computer-aided design (CAD) software. Fluid flow velocity profiles within the biocartridge were simulated for each model using the simulation package in Solidworks^®^. The parameters defined for the CFD are specified in Table 1.

**TABLE 1:** Initial conditions used for CFD and porous media CFD database definition.

Initial conditions	
Temperature (°C)	37°C
Pressure (Pa)	101,325
Gravitational force (m/s2)	9.81
Fluid	Water
Inlet flow rate	320.19 mL/min (5.3365e^−06^ m^3^/s)
Flow type	Laminar and turbulent
Wall thermal condition	Adiabatic
Boundary conditions	Inlet at uniform flow rate
	Outlet at environmental pressure
Global mesh	Refinement 3
Local mesh	Local meshes with different refinement levels located in inlet, outlet, 4-channel splitter, base plate, inner wall surfaces, metal surfaces, base plate mesh and top mesh areas

Porous media	
300 µm top cylindrical filter mesh	
Porosity	0.26
Permeability type	Unidirectional
Resistance calculation formula	Dependency on pore size
Pore size	0.0003 m
200 µm base plate mesh	
Porosity	0.204,649
Permeability type	Unidirectional
Resistance calculation formula	Dependency on pore size
Pore size	0.0002 m

The CFD analyses were calculated on liquid flow in a biocartridge; the hydrogel spheres containing the biomass were not included in the simulation.

### 2.3 Mean velocity value calculation from computational fluid dynamic (CFD) results

ImageJ software (Version 1.51j8) was used to calculate mean velocities in different height planes across the upright tubular biocartridge. For each plane, a colour is assigned for each velocity (defined as a range of speeds); the average area covered by each of the colours was then calculated and the mean velocity derived using Microsoft Excel software.

### 2.4 Monolayer cell culture and encapsulation of alginate encapsulated liver spheroids (AELS) or empty alginate beads (EAB)

HepG2 cells, originally obtained from ECACC Wiltshire, and subsequently rederived in GMP by Cobra Bio, were cultured and stored as previously described (Selden et al., 2017), using modified AlphaMEM medium (Supplementary Table S1). The Master Cell Bank (MCB) and a Working Cell Bank (WCB) were assessed for cell line identity and sterility was confirmed (Porton Down, Wiltshire and Vitrology, Scotland, United Kingdom). Initially, cells were cultured in triple-layered tissue culture flasks (T500, Nunc, Fisher Scientific, 10272721) and subsequently expanded into seven 10-layer cell culture flasks (CF10, Thermo Fisher Scientific, 140410) to achieve sufficient seeding cells (12–16 × 10^9^ cells) for encapsulation of 2.4–2.7 L microbead biomass, as previously described (Selden et al., 2017).

The encapsulation method was as previously described (Erro et al., 2013; Selden et al., 2017). Briefly, AELS (12–16 × 10^9^ cells) were resuspended in supplemented AlphaMEM media (Supplementary Table S1). 2% alginate (Manugel GMB, FMC Biopolymers) in 0.015 M HEPES, 0.15 M NaCl at pH 7.4, was mixed with the cells in a 1:1 ratio to achieve a final concentration of 2 × 10^6^ cells per mL in 1% alginate solution. Additionally, glass spheres (10–50 μm, Kisker) were added at a concentration of 1.3% w/v as a density modifier, to ensure an appropriate density of the alginate beads for a microgravity environment in both culture media and plasma. Alginate beads were formed with a multi-nozzle Jetcutter (GeniaLab^R^) using liquid, alginate and cells. The cell-alginate mix was delivered at 20 ± 1 mL/min. The alginate-cell solution, cut by horizontally rotating wires, led to the production of alginate/cell droplets falling into a polymerisation solution (0.204 M CaCl_2_ in 0.15 M NaCl pH 7.4) providing divalent calcium ion crosslinking and bead formation. After alginate bead production, excess calcium was removed by washing the beads five times with 3 L of supplemented medium (Supplementary Table S2). For production of EAB, the same process was followed using the Jet cutter as above, but without cells in the mixture (media composition described in Supplementary Table S2).

### 2.5 Culture of alginate encapsulated liver spheroids (AELS) after encapsulation and post-thaw recovery

The biocartridge design (B3) was filled with 2.4–2.7 L of AELS obtained from encapsulation, or AELS thawed from cryopreservation. The fluid inlet and outlet ports of the biocartridge were connected to the Single-use Bioreactor (SUB) with tubing to create a perfusion loop. The SUB consisted of a stirred tank with a bioprocess container (Thermo Fisher, Hyclone 100L SUB) and a controller unit (EZ Control, Applikon) with BioXpert SCADA software. Cell culture medium was pumped into the biocartridge through the inlet pipe, creating a microgravity environment. A peristaltic pump at 300–380 mL/min delivered culture medium through the biocartridge, expanding the AELS bed height 1.67-fold. Cells within alginate microbeads were cultured at a ratio of 1:46.4 (cell beads: cell culture medium volume) to enable cell proliferation into organoids.

During the AELS growth phase, fresh cell culture medium was replenished on days 4, 7, 9 and 11, at 80%, 60%, 70% and 80% respectively. AELS were harvested on day 11 or day 12 for testing or cryopreservation. Cryopreserved AELS underwent cryorecovery post thaw culture (PTC) for up to 4 days, with culture medium replenished on days 1 and 3 at 80%. AELS were harvested on day 4 of post-thaw recovery culture.

The bioreactor-controlled AELS culture conditions were maintained using proportional–integral–derivative (PID) mechanisms, maintaining a temperature of 37°C and a pH of 7.4. Fluid dissolved oxygen levels pre- and post-biocartridge were measured using polarographic dissolved oxygen sensors. Oxygen saturation was maintained between 15% and 35% to avoid hypoxia or hyperoxia conditions. Oxygen concentration in the bioreactor reservoir was gradually increased by 1% increments, reaching a maximum of 35%, to compensate for cellular consumption. When a 35% dissolved oxygen concentration in the reservoir was insufficient to maintain the outlet concentration above 15%, additional oxygen was supplemented internally to the biocartridge using the gas-permeable silicone tubing coils positioned at the periphery and within the core of the fluidised bed. The supplementation of AlphaMEM media is described in Supplementary Table S3. Additionally, on days 9 and 11 of culture, an amino acid mix of phenylalanine, cysteine, leucine, isoleucine and methionine was used to supplemented medium to prevent amino acid depletion (Erro et al., 2013).

### 2.6 Equilibration time and bed fluidisation studies using EAB or fully-competent AELS in the new biocartridge (B3)

For estimating the equilibration time, 2.5 L of EAB were inserted into the single-use biocartridge (B3) and a recirculation loop was created with a peristaltic pump and a 10 L reservoir. The system was primed with 10 L of 0.15 M NaCl, 2 mM CaCl_2_ and actively recirculated. The EAB bed was expanded to 1.67-fold with a constant flow rate of 358 mL/min. Bromophenol blue (16 mL of 0.04 mg/mL) was injected at the inlet fluid port. The fluid mixing profile was determined by collecting samples from the outlet port at timed intervals, and absorbance was measured at 592 nm.

For EAB fluidisation studies, once the circuit was fully primed, fluid recirculation was stopped for 30min and the vertical height of the settled bed of beads (H_0_) was measured. The fluid recirculation was restarted, and bead bed heights measured at increasing flow rates once the expanded bed reached a stable height (H_f_) at each new flow rate. For estimating fluidisation using AELS, the same principle was used but applied during the proliferation phase and the loop consisted of a single-use bioreactor containing 110 L of culture media and the single-use biocartridge filled with 2.7 L of AELS.

### 2.7 Cryopreservation and thawing of alginate encapsulated liver spheroids (AELS)

The cryopreservation and thawing protocols were based upon previous work, Brookshaw et al. (2024). For cryopreservation, on the day of harvest (day 11/12 of fluidised dynamic culture, FDC) 2.4–2.5 L of AELS were cooled to 5°C and left to settle in a 5 L sterile vessel. The AELS were mixed 1:1 with the cryoprotectant solution over a 5 min period and left to settle. 50% of the CPS supernatant volume was then removed, the AELS in residual CPS were pumped into three cryobags (CS2000, Origen), covered and sealed using an overwrap bag (Origen, OW2436), and cooled using a controlled rate freezer (Kryo750, Planer Ltd.). Delta7 software monitored the ambient temperature in the cryo-chamber during cooling. A cooling profile of a rate of −1°C/min for 1 h was set, followed by a 2 h hold at −50°C, and subsequent cooling rate of −0.5°C/min until the samples reached a temperature between −100°C and −120°C. Thereafter, samples were transferred to vapour phase of liquid nitrogen storage until use.

During thawing, three cryobags (CS2000, Origen) were transferred from vapour phase liquid nitrogen to a-80°C freezer for 1 hour to avoid cracking of the cryo-bags during warming. The cryobags were then submerged in a 37°C water bath and rotated gently until the last ice crystal melted, over the course of 12–14 min, and kept on ice until washing. In a Class II microbiological safety cabinet, the cryo-overwrap was cut and removed, and the contents of the three bags were poured into a 200 µm stainless steel cylindrical mesh contained in a large stainless-steel vessel. The CPS was drained, and the biomass washed twice at 4°C using decreasing concentrations of glucose, 1M and 0.5 M respectively (Sigma-Aldrich, G5767) in DMEM in a 1:2 bead to media ratio. Finally, the AELS in plain DMEM (25 mM glucose) in a 1:2 ratio were washed at room temperature, resuspended in cell culture medium and loaded into the biocartridge for post thaw culture (PTC). Chemicals used during cryopreservation are listed in Supplementary Table S4.

### 2.8 Cell viability assessment, image analyses and AELS component cell enumeration

Methods of assessment included microscopy, fluorescent dye staining, and metabolic profiling as previously published (Figaro et al., 2015; Van De Kerkhove et al., 2002; van der Hilst et al., 2009). Chemicals and materials used for viability and cell counting are listed in Supplementary Table S5. For fluorescent dye analyses, 250 µL of AELS beads were washed twice in 1 mL of phosphate buffered saline (PBS), resuspended in 0.5 mL of PBS and stained using viability dyes, fluorescein diacetate (FDA), for live cell staining and propidium iodide (PI), for dead cell staining for 90 s. After staining the AELS were washed in 1 mL of PBS and resuspended in 0.5 mL of PBS. Images of five fields were captured using a Nikon TE200 fluorescent microscope with a Nikon DS-Fi1c camera at ×4 magnification. The cells were visualised using an excitation filter of 510–560 nm and an emission filter of 590 nm for PI-stained cells, at exposures of 800 ms. For FDA-stained cells, an excitation filter of 465–495 nm and an emission filter of 515–555 nm was used, at exposure of 100 ms. The percentage viability was quantified using NIS elements imaging software, as previously described (Selden et al., 2017).

For component cell enumeration, 1 mL of AELS samples (n = 5) were placed in a 15 mL conical tube. 5mL of a 16 mM EDTA solution (0.15 M NaCl, Fisher Scientific, pH7.4) was added to chelate the calcium from alginate and liberate the spheroids. The resulting cell solution was centrifuged at 4000 *g* for 10 min. After discarding the supernatant, cells were resuspended in the required volume of PBS^−Ca-Mg^ (Gibco, 14190), and nuclei released using passage through a 21G needle (BD, 304432). A volume of 0.5 mL containing the cell suspension was lysed using reagent A (Chemometec, 910-0003), vortexed for 10 s and then reagent B (Chemometec, 910-0002) was added for cell stabilisation and mixed for 5 s (Selden et al., 2017). A nucleocounter (Chemometec, NC-100 or NC-200) was used to measure cell number by nuclei counting, possible as the cells are mononucleate.

### 2.9 Glucose consumption and lactate production

Glucose consumption and lactate production were quantified by the GM-7 Micro-stat analyser (Analox). Glucose oxidase or lactate oxidase enzymes were used for determining the concentration of glucose or lactate analytes in the culture media, respectively. Quantification is calculated from the oxygen consumption rate during oxidation and depends on the concentration of analyte.

### 2.10 Protein synthetic function: alpha-fetoprotein (AFP) ELISA

AFP protein concentration in media during AELS culture was measured by enzyme-linked immunosorbent assay (ELISA). Primary (Abcam, Ab10071) and secondary horseradish peroxide (HRP)-linked antibodies (Abcam, ab10072 OR Generon, CSB-PA09987B0Rb) were used. For quantification, the standard curve was performed using human AFP antigen (AppliChem, A6935) with a range of 200 ng/mL to 12.5 ng/mL.

### 2.11 Analysis of alginate bead sizes

Phase contrast images of AELS and EAB were utilised to determine the average diameter using NIS Elements software. For analyses, a five-point ellipse was created to outline the alginate beads, calculating the diameter.

### 2.12 Statistical analyses

Statistical analyses were conducted using GraphPad Prism 10. Data were first assessed for normality using Shapiro-Wilk test. For comparison between two groups, Welsch t-test was used for normally distributed data, and for not normally distributed samples, Mann–Whitney U test was applied.

## 3 Results

### 3.1 Computational fluid dynamic modelling results

**FIGURE 1:**
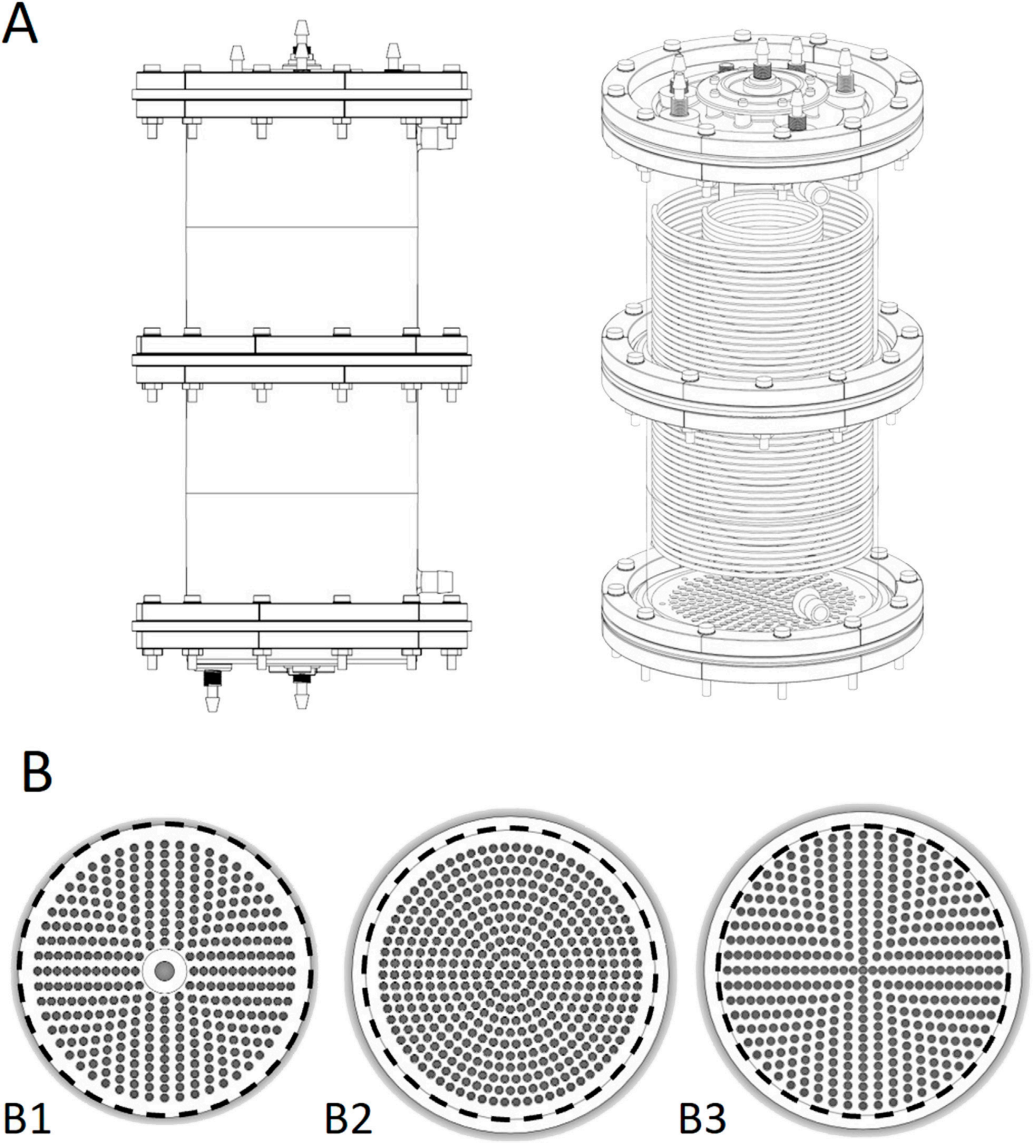
**(A)** CAD drawing of the single use biocartridge (top left) and 3D rendering of CAD design (top right). **(B)** base plate designs for biocartridges. Bottom left to right: B1, B2 and B3 base plates. The numbers and patterns of the penetrating holes were amended to understand their impact on fluid flow distribution. The inner diameter of the area exposed for fluid flow in the biocartridge in B1, B2 and B3 is 150 mm (represented by black dashed line). The outer dimension of the B1 was 150mm, and 164 mm for B2 and B3. Therefore, a circular line was drawn at 150 mm for figures B2 and B3 above for illustrative purposes.

During this study, a single-use biocartridge (B3) (Figure 1A) was developed as a constituent of the HepatiCan™ Bioartificial Liver (BAL) device, using medical grade components suitable for clinical use. Whilst the reusable biocartridge (B1) was made of stainless steel, Peek, glass, silicone gaskets and rubber O-rings, B3 was designed to be manufactured by injection moulding of Cyclic Olefin Copolymer (TOPAS), stainless-steel, silicone O-rings and gaskets. Two different disposable designs (B2 and B3) were investigated for suitability *via* CFD, with simulations indicating that B3 was the optimal final design. This was then produced by injection moulding using bespoke moulding tools.

The disposable prototypes (B2 and B3) were designed to mimic the reusable cartridge (B1) from a functional perspective. The flow distributor plate thickness of B1 was 17 mm. The thickness of the B2 and B3 distributor plate designs was 5 mm to reduce cost of goods without compromising functionality. All the base plate designs had hole sizes of 3.5 mm (Figure 1B).

The primary difference among the base plates (in designs B1, B2, and B3) was the pattern and number of orifices in each design. B1 contained 354 holes arranged in a cross-shaped pattern which was completed by filling the gaps between crossed axis filled in a circular manner (Figure 1B). The B3 design had a similar pattern to B1, but with 421 holes. The B2 design had a base plate with 352 holes arranged in a circular pattern with 5 mm pitch between holes. Both B1 and B2 plates contained a non-functional central region, due to a supporting rod located vertically under the base plate between the 4-channel flow splitter and the bottom of the base plate. B3 did not contain this rod. Additionally, for B1 and B2 the holes did not reach to the perimeter of the cartridge, but did for B3 (Figure 1B).

### 3.2 Horizontal and vertical cross-sectional velocity modelling profile comparisons from CFD analysis

**FIGURE 2:**
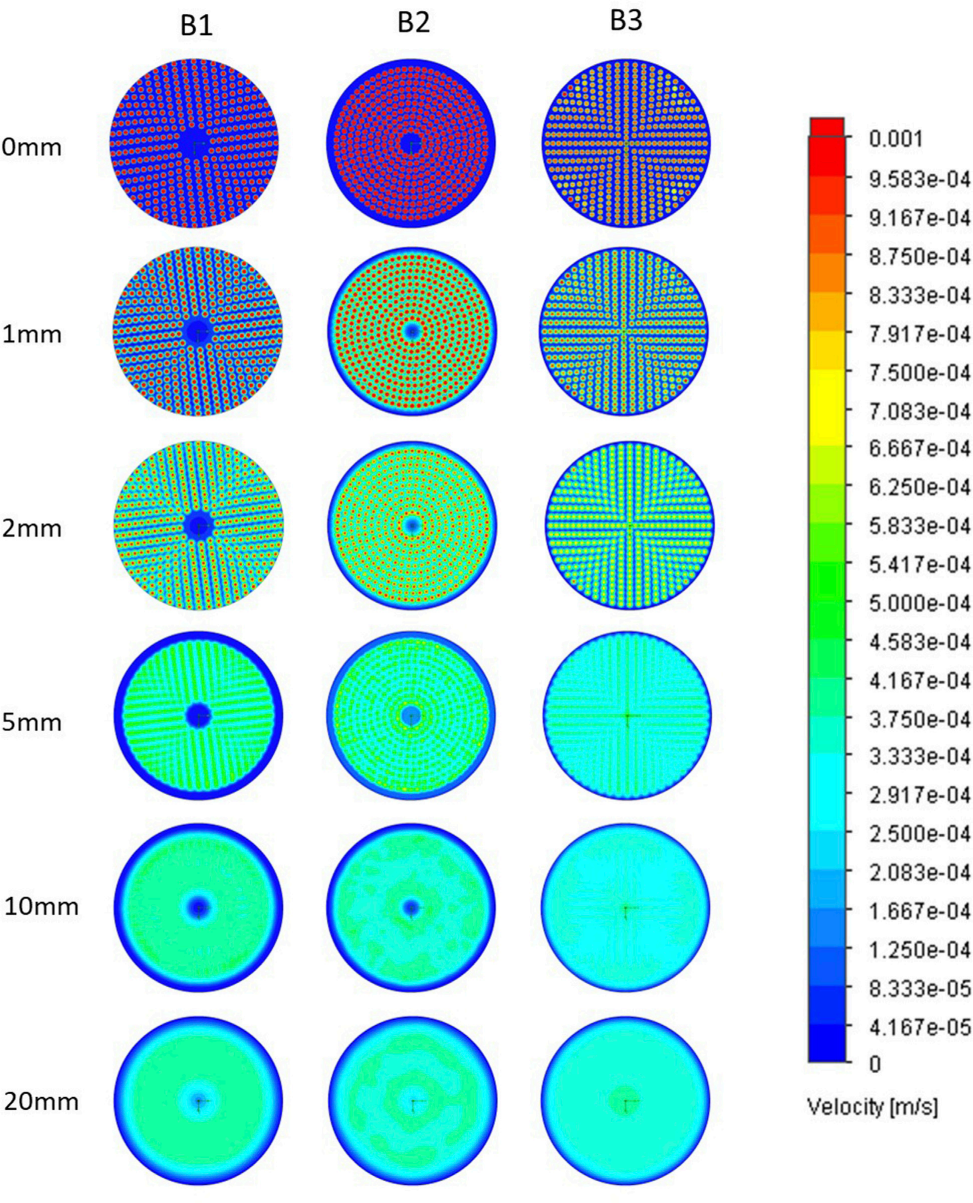
Horizontal plane fluid velocity plots at ascending heights above base plate in B1 (left column), B2 (central column) and B3 (right column) designs. Each of the images in a row represents a horizontal cross-sectional of a biocartridge at different positions above the base plate mesh. Computational fluid dynamics (CFD) results are presented using colour gradients to illustrate varying velocity ranges.

**FIGURE 3:**
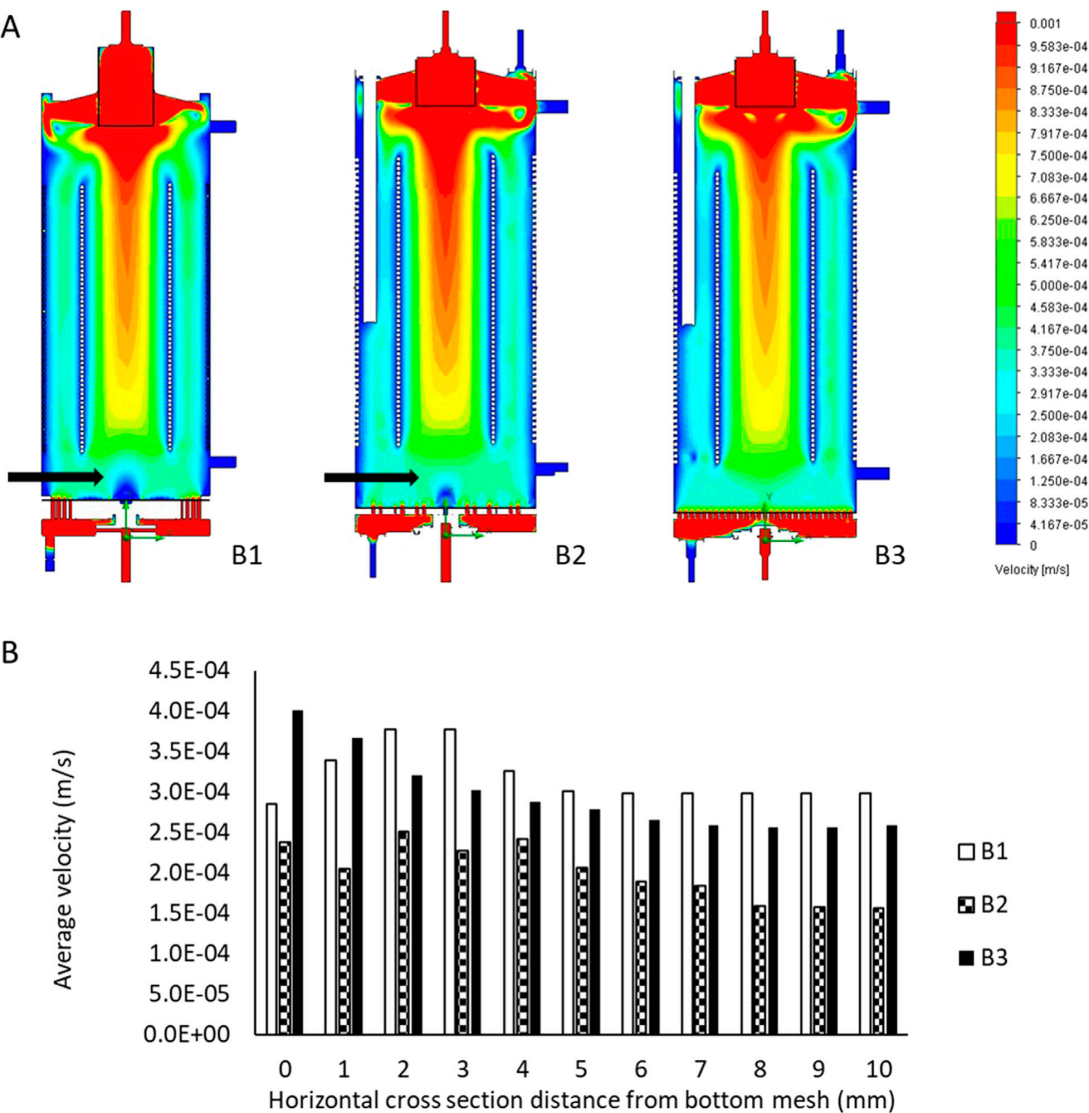
**(A)** Flow regimes inside the B1 (left), B2 (middle) and B3 (right) biocartridge designs. Each of the images represents a vertical cross-section at the central core of the biocartridge. Black arrows highlight slow velocity areas above bottom base plate mesh. CFD results are presented using colour gradients to illustrate varying velocity ranges. **(B)** mean fluid velocity comparisons for the reusable biocartridge model (B1), and the disposable biocartridge designs B2 and B3 at different horizontal cross-sectional planes, moving upwards in the biocartridges above the base plate. Data collected from Modelling using CFD and averages calculated by further image analyses with Image J.

Horizontal cross-sectional velocities in thein biocartridge design B3 were closer to the original B1 than to B2 within the first 2 mm above the base plate. In contrast, the B2 design showed faster velocities in those areas (Figure 2). In addition, B3 demonstrated more uniform velocity distribution and fewer slow velocity zones compared to both the B1 and B2 designs, in the 5–10 mm cross-sectional areas above the base plate (Figure 2). These slow-velocity zones (represented by dark blue colour) were primarily located at the periphery and central areas, with velocities ranging from 0 to 4.17 × 10^−5^ m/s. At a height of 20 mm above base plate, all three designs showed similar homogenous flow distributions. Vertical cross-sectional velocity profiles were comparable between the three biocartridge designs (Figure 3A). However, some small differences were observed in both B1 and B2, in comparison to B3, such as slow velocity areas just above the base plate mesh central area. This was due to the total blockage of the holes in the central areas of the base plate for the design of B1 and B2, and contributed to a better homogeneity in the hydrodynamic conditions above the base plate filter in B3. Furthermore, a noticeable increase in fluid velocities was observed at the top centre of all biocartridges, likely due to the narrowing of the outlet channel.

### 3.3 Mean velocity results of B1, B2 and B3 designs

The mean velocities for the reusable biocartridge (B1) increased from 2.86 × 10^−4^ m/s at the top of the base plate to 3.77 × 10^−4^ m/s at 3 mm above the base plate (Figure 3B). The mean velocity then decreased to reach 2.98 × 10^−4^ m/s at 6 mm above the base plate, maintaining similar mean velocity values from 6mm to 10 mm above the base plate. In the B2 design, mean velocity fluctuated between 1.56–2.5 × 10^−4^ m/s; mean velocities in the analysed section areas were slower compared to B1 and B3. The B3 disposable biocartridge design showed the highest initial mean velocity, immediately at base plate mesh cross-sectional area (4.02 × 10^−4^ m/s at 0 mm distance from base plate), which decreased over the cross-sectional areas from 0 to 7 mm height to 2.59 × 10^−4^ m/s. A stable mean velocity profile was obtained from 7mm to 10 mm for B3; at 5–10 mm for B1 and at 8–10 mm for B2 (Figure 3B).

### 3.4 Estimating the circulatory equilibration time and fluidisation parameters using EAB and AELS

**FIGURE 4:**
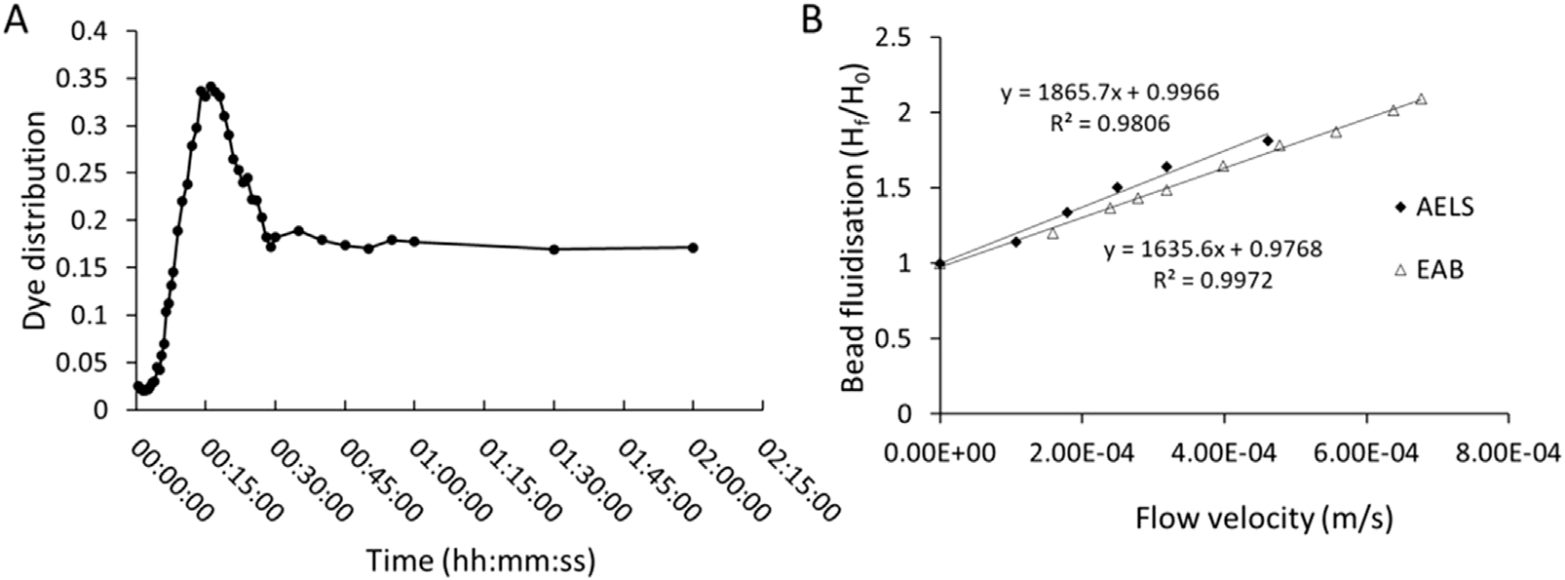
**(A)** mixing pattern over time in the single-use biocartridge (B3) at 1.67-fold expanded bed (at fluid flow of 358 mL/min) after injection of Bromophenol blue dye. Absorbance measured at 592 nm. **(B)** comparison of bed expansion (Final bed height, H_f_/Initial bed height, H_0_) in biocartridge using empty alginate beads (EAB) or alginate encapsulated liver spheroid (AELS) beads (at culture day 6) as a function of increasing superficial velocities (u).

An important parameter in dynamic environments within biocartridge circuits is the mixing time, as this represents the capacity to reach equilibrium and homogeneity in distribution of the mobile fluid phase supporting the bed of alginate beads. Dye dilution analysis showed that bromophenol blue concentration peaked 17 min after injection and gradually decreased, reaching equilibrium at 60 min. The initial high peak represents circulating time, and the time taken to achieve a stable level represents the mixing time (Figure 4A). Fluidised bed bioreactors follow the Completely Mixed Flow (CMF) model, rather than the plug model, for the theoretical calculation of mixing time (Andrews, 1988; Godia and Sola, 1995). Hence, the time a particle stays in the system, or hydraulic retention time (θ) is calculated by the following formula (Equation 1):

θ=V/Q (1)

V represents the volume and Q the volumetric flow rate. This represents the characteristic time of the system and for recirculation studies, the recirculation time (τ), where (θ = τ). The mixing time t_m_ is related to τ, assuming a 95% mixing state. For the calculation of the mixing time the following formula is given (Equation 2):

$$t_{m,95}=3\tau$$ (2)

For the calculation of these parameters in the single-use biocartridge B3, the channelling effect of the initial stagnant volume of the reservoir needs to be considered (Fogler and Brown, 2006). Out of the 10 L of the total recirculating fluid volume, the biocartridge volume contained 64.6% (i.e. 6.46 L) at 358 mL/min flow rate, thus the theoretical recirculation time was θ = 18.1 min. The mixing time was calculated from Equation 2, resulting in 54.1 min. The empirical values (Figure 4A) for recirculation time (16 min) and mixing time (60min) were well-matched to theoretical values. Additionally, the fluidisation and alginate bead bed expansion of AELS and EAB were empirically determined (Figure 4B). For AELS an initial 1.14-fold bed expansion was achieved at 1.08 × 10^-4^ m/s linear velocity and followed a linear pattern with increasing velocities: achieving 1.64-fold at 3.19 × 10^-4^ m/s and a 1.81-fold expansion at 4.61 × 10^-4^ m/s velocity. EAB followed a similar fluidisation linear pattern to those loaded with cell spheroids, achieving a 1.75-fold expansion at 4.61 × 10^-4^ m/s velocity. The ratio (H_b_/D) of settled bed height (17.5 cm for 2.7 L of alginate beads) to biocartridge diameter (15 cm) was 1.17. This parameter influences the fluid velocity and mixing in the bioreactor. There were no statistical differences in average alginate bead diameters for either the AELS or EAB used during fluidisation experiments, which were 515.27 ± 90.9 μm (n = 188) and 515.82 ± 92.3 μm (n = 100) respectively (Mann-Whitney U test, P value = 0.91).

### 3.5 Cell density comparison between the reusable biocartridge (B1) and disposable biocartridge (B3)

**FIGURE 5:**
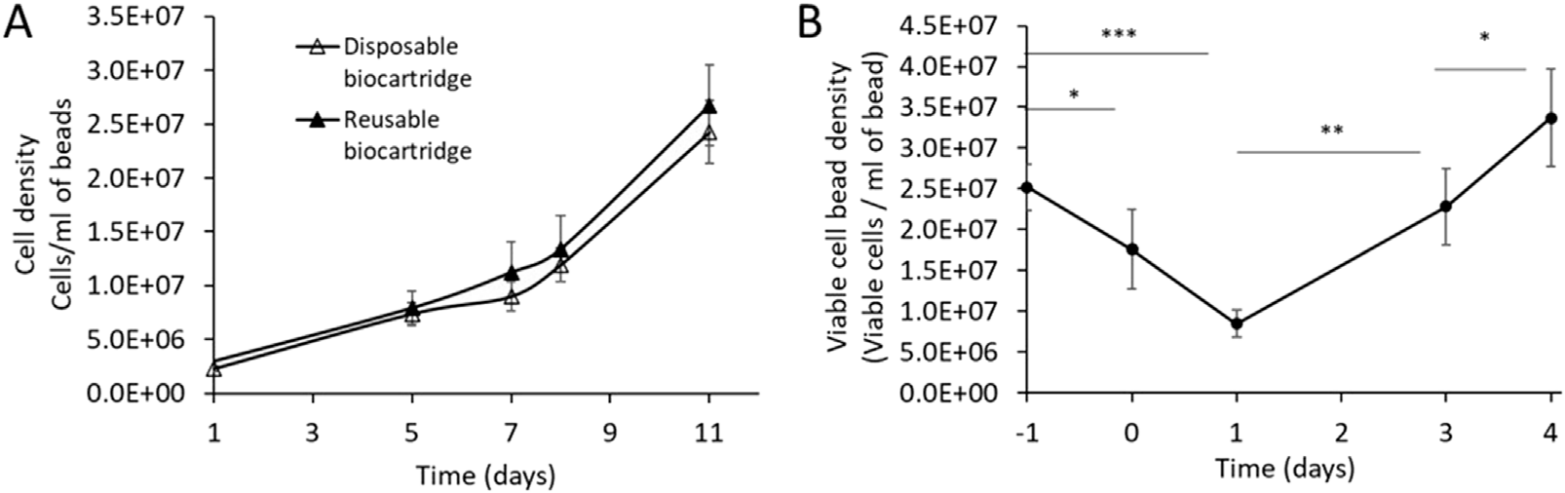
**(A)** cell proliferation profile during cell growth phase runs in disposable (B3 design, n = 4) *versus* reusable biocartridge (B1 design, n = 15). Data shown as mean ± SD. For statistical analysis, data were normally distributed and compared using Welch’s t-test on day 11. There was no significant difference P = 0.149. **(B)** viable cell bead density of AELS biomass recovery after cryopreservation in the disposable biocartridge, over 4 days (days 0–4, n = 4). Day −1 represents cell density at cryopreservation. Data shown is mean ± SD. For statistical analysis, data were normally distributed and compared using Welch’s t-test. Statistical significance was denoted as follows: *p < 0.05, p < 0.01, ***p < 0.001, ****p < 0.0001.

No significant differences (day 11, Welsch’s t-test, P = 0.149) were observed (Figure 5A) in the cell density of AELS grown under FDC conditions between the disposable biocartridge (B3) and the original biocartridge (B1). The viability of the AELS remained consistently high throughout the growth phase, never falling below 98% for the disposable biocartridge B3 (n = 4) and 91% (n = 14) for B1. These data verify that the disposable biocartridge (B3) is an effective replacement for the reusable biocartridge (B1). The following AELS data were obtained using the disposable biocartridge (B3).

### 3.6 Viable cell number and viability of the liver cell spheroids in the disposable biocartridge (B3) during growth phase

Using the B3 design, AELS reached 24.27 ± 2.9 × 10^6^ cells per ml of settled bead volume after an 11-day continuous FDC culture period, following a sigmoidal curve and remaining in exponential phase of growth. This equates to an approximate total biomass load of 6.88 × 10^10^ ± 1.0 × 10^10^ cells per production cycle at day 11. The viability of the biomass during the FDC period remained consistently high, above 98% throughout the growth period.

### 3.7 AELS biomass performance during the growth phase

**FIGURE 6:**
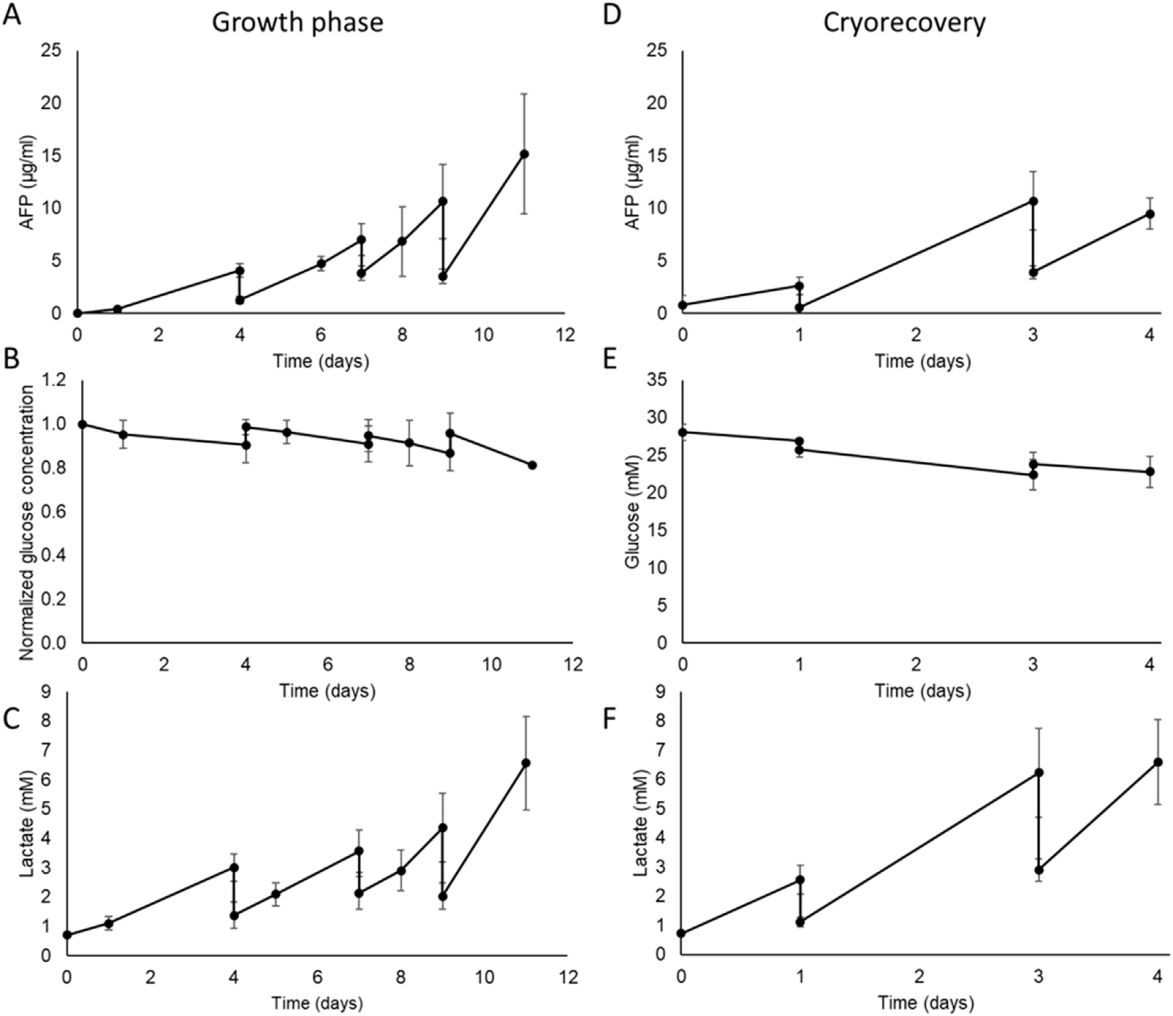
Metabolic characterisation of the AELS under FDC conditions in the disposable (B3) bioreactor biocartridge cultured during growth phase (Left-hand panels) and post-thaw culture phase (Right hand panels). Data shown is n = 4, mean ± SD. **Growth phase: (A)** AFP concentration (µg/mL) in cell culture media during AELS cell growth phase. **(B)** normalized glucose concentration in cell culture media during cell growth phase of AELS. **(C)** lactate (mM) concentration in cell culture media during AELS cell growth phase. **Recovery phase after cryopreservation PTC: (D)** AFP concentration (µg/mL); **(E)** glucose concentration (mM); **(F)** lactate concentration (mM); in cell culture media during AELS cell recovery phase. AELS after cryopreservation show similar metabolic trends to those seen with fresh AELS.

Alpha-fetoprotein (AFP), used as a surrogate for liver spheroid protein synthesis, was continuously produced throughout the AELS growth culture period (see Figure 6A), reaching a peak concentration of 15.15 ± 2.9 μg/mL on day 11 in the culture media. The cyclical decreases observed in AFP concentration shown in Figure 6 were a result of the scheduled partial replenishment of the cell culture media during planned media changes.

The glucose concentration in the cell culture media decreased gradually throughout the growth phase (Figure 6B), representing glucose consumption. As expected, glucose levels rose on the days of medium supplementation (days 4, 7, and 9), when fresh glucose-containing cell culture media was added by an 80%, 60% and 70% medium change, respectively. There was a 9% drop in glucose concentration by day 4; an 8% drop between days 4 and 7, and days 8 and 9, and a 15% drop between days 9 and 11. Glucose consumption was sustained over time. The glucose concentration remained at an adequate level for cell proliferation throughout the growth period (>22 mM glucose), supported by the medium replenishment regime.

With AELS, lactate concentration in the cell culture media increased over time as expected due to the aerobic glycolytic consumption of glucose (Warburg effect) by HepG2 cells (Figure 6C). Inversely compared to glucose concentration, lactate levels decreased on days 4, 7, and 9 after dilution with fresh medium. Lactate concentration peaked on day 11 at 6.57 ± 1.6 mM. There was a sustained production of lactate over the entire growth period. The highest lactate production (15.77 ± 5.2 µmol per million cells per day) occurred on day 1, significantly higher than the levels on days 7, 8, and 11, which were 3.06 ± 3.2, 1.59 ± 1.4, and 1.88 ± 1.7 µmol per million cells per day, respectively (Supplementary Figure S1). Taking both glucose consumption and lactate production over the entire FDC growth phase, they demonstrate aspects of high metabolic activity within the biocartridge.

### 3.8 Cell viability and cell numbers during recovery of AELS after cryopreservation

B3 design was used to test post-cryopreservation FBB culture of thawed biomass. Viability of AELS before cryopreservation was 98.63% ± 0.3% (n = 4), decreasing by 17% after thawing (day 0) and a further 18% by 24 h of culture, reaching the lowest viability of 67.05% ± 7.4% on day 1. By day 4 of re-culture after cryopreservation, viability returned to 94.66% ± 5.4%, not significantly different from the pre-freeze value (Welch’s t-test, P = 0.268).

During PTC, the viable cell density of AELS dropped significantly (Welch’s t-test, P = 0.043) on thawing (day 0) from an initial value of 25.2 ± 2.8 × 10^6^ cells per ml of alginate beads to 17.6 ± 4.8 × 10^6^ (Figure 5B). On day 1 the value reduced to the lowest viable cell density of 8.5 ± 2.8 × 10^6^ cells per ml of alginate beads (Welch’s t-test, P = 0.0002) post cryopreservation. By day 3 of PTC, the viable cell density did not significantly differ (Welch’s t-test, P = 0.42) from pre-freeze levels, and viable cell density increased to 33.70 ± 6.0 × 10^6^ cells/mL of alginate of beads by day 4 PTC.

A total biomass yield of 6.34 ± 0.9 × 10^10^ viable cells was cryopreserved and recovered during 4 days of PTC, achieving a total cell number of 5.74 ± 1.2 × 10^10^ cells on day 3 and 8.48 ± 1.6 × 10^10^ cells on day 4. There was no significant difference in the cell number before cryopreservation and day 3 PTC (Welch’s t-test, P = 0.4631).

### 3.9 Physical characteristics and metabolic performance in AELS after cryopreservation

Protein synthesis, as estimated by measuring Alpha-fetoprotein concentration in the cell culture medium, increased during the PTC, reaching peaks of 2.62 ± 0.8 μg/mL, 10.70 ± 2.7 μg/mL, and 9.49 ± 1.5 μg/mL after 1, 2, and 4 days of culture, respectively (Figure 6D). The observed drops in AFP on days 1 and 3 resulted from routine fresh medium replacement days 1 and 3. Glucose concentration exhibited a gradual decrease throughout the culture period from 28.06 ± 1.4 mM to 22.81 ± 2.1 mM by day 4 of culture, as expected during cell proliferation, and stabilised by the media changes (Figure 6E). Lactate concentration increased during the cryo-recovery culture process (Figure 6F). An initial peak value of 2.57 ± 0.5 mM was reached on day 1 of the culture. Subsequently, maximum values of 6.23 ± 1.5 mM and 6.59 ± 1.5 mM were reached on days 3 and 4 respectively. The daily production of AFP and lactate showed a linear accumulation over time (Supplementary Figure S2). The average diameter of AELS at the end of the growth phase was 526.70 ± 95.46 μm (n = 369) and on recovery day 3 after cryopreservation was 535.80 ± 124.99 μm (n = 315). These data showed no significant difference in average diameter between both conditions (Mann Whitney U test, P value = 0.07), maintaining bead size over time.

## 4 Discussion

With the aim of developing an efficient bioartificial liver treatment as a potential alternative to liver transplantation, a single-use disposable biocartridge (B3), a component of HepatiCan™ BAL, was produced and evaluated. Computational fluid dynamic (CFD) data demonstrated homogeneity and adequate hydrodynamic conditions for AELS biomass production, and an improved performance compared to the reusable research biocartridge (B1) due to the lack of dead zones for flow. The B2 design had faster velocity points in the region located 0–2 mm above the base plate. B2’s increased hydrodynamic conditions could result in higher shear forces impacting microcapsule integrity of AELS (Lu et al., 2016). The perfusion velocities observed in B3 on the first 2 mm above the base plate, comparable to B1, should minimise microbead damage during the culture phase of AELS and during PTC. The B3 design mimicked our well-characterised reusable biocartridge B1 (Erro et al., 2013; Selden et al., 2017; Selden et al., 2013). The hole pattern played a crucial role in replicating hydrodynamic conditions of the original design. Additionally, B3 contained more functional holes resulting in a more uniform hydrodynamic condition across the fluid column above the base plate. B3 enhanced fluid particle homogeneity and overall quality of fluidisation (Windows-Yule et al., 2020). In turn, this minimises the formation of “dead zones” or “stagnant areas” (Zhang et al., 2010). Some other systems do not include a distributor, to avoid channelling effects (generated by preferential paths and uneven speed points), achieving functionally performing bioreactors (Agu and Moldestad, 2018). It is generally agreed that a distributor plays a key role in the success of fluidised bed bioreactors (Sobrino et al., 2009; Wormsbecker et al., 2007; Zheng and Zhu, 2003). It is important to emphasise the presence of a double distributor in our biocartridges, a horizontal 4-way flow splitter at the entrance and a perforated base plate above it. B3 faster mean velocity patterns on the horizontal cross sections, with slower mean velocities observed for the B2 design were due to both heterogeneous flow dynamics and a different base plate hole pattern.

Cell proliferation was comparable between B3 and B1, with the enhanced flow dynamics in B3 likely promoting more uniform cell growth and better cell viability, with increased mass transfer, and fewer stagnant zones. Mass transfer values (for B12 and albumin) for alginate beads in fluidised bed bioreactor systems have been determined previously (David et al., 2004; Mendonça da Silva et al., 2020). Size of the microspheres, composition of hydrogel and size of diffusing particles can all influence mass transfer (Gautier et al., 2011; Sun et al., 2018). Our single-use biocartridge mass transfer values were expected to align with the findings reported by Mendonca *et al.*, where similar-sized alginate beads made of the same hydrogel product were tested, at a different scale, under FDC regime (Mendonça da Silva et al., 2020).

In conjunction with the hydrodynamic conditions of the liquid-phase fluid flow results, one should understand the solid-phase microgravity conditions - the upward and downward movement of the alginate beads, created in response to FDC within the biocartridge. Previous studies (Legallais et al., 2000; Naghib et al., 2017) have characterised fluidisation of alginate beads in fluidised-bed bioreactors. The potential of fluidisation-based biocartridges as bioartificial liver devices in a plasma milieu have also been theoretically and empirically validated (Doré and Legallais, 1999; Figaro et al., 2015; Legallais et al., 2000). A linear microbead bed expansion was observed in this study exhibiting the expected performance and confirming adequate dispersion of the fluid, consistent with other similar systems (Legallais et al., 2000; Mendonça da Silva et al., 2020). Empirical values for recirculation and mixing times agreed with theoretical values, confirming the efficiency of the new design. The mixing time ensures that cell spheroids are exposed to similar conditions, such as nutrients and oxygen levels, and recirculation time reflects how quickly the nutrients and waste products circulate in/out of the biocartridge. Optimising both values in a FBB are determining factors contributing to an ideal environment for high cell density and functionality. Microscopy data for live-dead cell staining indicated adequate mass transfer for AELS formation as no dead cell regions or poor cell proliferation were observed in central areas of the alginate beads, as previously proven (Erro et al., 2013). During this study an alginate bead diameter size of 526.70 ± 95.46 μm after the growth phase was comparable to the value after cryopreservation (535.80 ± 124.99 μm), suggesting stability of the microspheres after cryopreservation. These values closely match our previous findings of 566 ± 74 μm (Erro et al., 2013) and 537 ± 72 μm (Selden et al., 2017) at the end of the growth phase, enabling comparison with AELS studies on the previous reusable biocartridge UCLBAL (HepatiCan™ predecessor).

In a previous study on the UCLBAL, an oxygen consumption of 0.153 fmol/cell/sec during day 12 AELS 3D microgravity culture was calculated (Selden et al., 2017) using oxygen diffusivity values from Gerontas et al. (2009) and experimental data from the fluidised bed bioreactor. This value was higher than previously reported value of 0.038 fmol/cell/sec (Erro et al., 2013), which may be attributed to differences in the hydrogel properties and higher cell densities using the same biocartridge. The latter value was more in accordance with oxygen consumption rates reported for the AMC bioreactor (0.0166 fmol/cell/sec) (Poyck et al., 2007). In contrast, Mueller *et al.* found a five-to-ten-fold lower values of 0.0028 fmol/cell/sec for human primary hepatocytes cultured in hollow-fibre reactors (Mueller et al., 2011). The higher oxygen demand per cell observed for our biomass, compared to other systems, may indicate a faster proliferation rate and a preference for an aerobic pathway.

During both PDC and PTC cultures, sustained biological function was observed, including continuous glucose consumption, lactate accumulation, and AFP production, a protein specific to this cell type, consistent with our previously published data (Erro et al., 2013; Selden et al., 2017; Selden et al., 2013). High levels of endogenous proteins (e.g., albumin and alpha-1-antitrypsin) present in fresh frozen plasma (FFP) and used as a media supplement, could interfere with accurate measurements. Instead, alpha-fetoprotein (AFP) was selected as a marker of synthetic function as it is absent in healthy plasma (FFP), but produced by HepG2 cells. Our studies have demonstrated that AFP secretion by conditioned HepG2 cells strongly indicates continuous protein synthesis by AELS (Massie et al., 2011; Selden et al., 2017; Selden et al., 2013).

Several BALs tested in the literature, in the (pre)clinical setting, differ in the technology configuration: the main types use hollow fibre technology: ELAD (Ellis et al., 1996), HepatAssist (Demetriou et al., 2004), MELS (Sauer et al., 2003), SRBAL (Li et al., 2018); and perfused matrices: BLSS (Mazariegos et al., 2001; Patzer et al., 1999) and AMC-BAL (Poyck et al., 2007; Van De Kerkhove et al., 2002). The most explored *ex vivo* BAL devices to date were based on cells entrapped in hollow fibre cartridges (Li et al., 2018; Mullon and Pitkin, 1999; Sauer et al., 2003; Sussman et al., 1994). Although, widely used and with benefits such as high cell density within tightly packed fibres, long-term stability and low shear stress, hollow fibre cartridge design exhibits several drawbacks compared to fluidised bed bioreactor (FBB) technology. One disadvantage is selective channel fluid flow, with plasma following the easiest route during treatment. This results in poor, non-homogenous fluid exchange between the cells and the environment, thus sub-optimal treatment conditions. The improved treatment efficiency due to higher mass transfer without pore blockage of Fluidised Bed Bioreactor devices, hosting encapsulated liver spheroids, confers technical superiority over hollow fibre devices, for Bioartificial Liver therapies (Fane and Fell, 1986; Macdonald et al., 2001; Moussy, 2003; Stressmann and Moresoli, 2008). Despite all the limitations for optimal *ex-vivo* treatment, until now these hollow fibre technologies have predominated, due to ready availability of dialysis cartridges and low cost, but with little emphasis on functional performance at clinical scale. Supplementary Table S6 describes earlier experimental systems for liver support created before 2015, none of which are in commercial use (Demetriou et al., 2004; Duan et al., 2007; Ellis et al., 1996; Li et al., 2018; Mazariegos et al., 2001; Millis et al., 2002; Patzer et al., 1999; Poyck et al., 2007; Sauer et al., 2003; Van De Kerkhove et al., 2002). In the past decade, updated approaches to liver support have been developed, listed in Supplementary Table S7 along with older existing BALs clinically tested (Chen et al., 2019; Duan et al., 2007; Ellis et al., 1996; Glorioso et al., 2015; Li et al., 2018; Millis et al., 2002; Opie et al., 2024; Selden et al., 2017; Selden et al., 2013; Thompson et al., 2018; Wang et al., 2023).

Preclinical studies on the HepatiCan™ predecessor (UCLBAL) have demonstrated therapeutic promise of the device in large animal models of ischaemia-induced acute liver failure (Selden et al., 2017; Selden et al., 2013). The functional efficiency of the alginate encapsulated liver spheroids (AELS) has also been assessed *in vitro* for the expression of a panel of phase I and phase II detoxifying enzymes, metabolic activity and synthetic function (Erro et al., 2013). Albeit lacking the urea cycle to metabolise ammonia to urea, HepatiCan™ showed decreasing ammonia levels in preclinical studies (Selden et al., 2013). Additionally, the bioartificial liver biomass increased bilirubin conjugation, synthesised and secreted proteins (albumin, alpha-1-acid glycoprotein, fibrinogen, prothrombin and alpha-1-antitrypsin) and restored clotting parameters (Selden et al., 2013). These preclinical studies provide strong support for the feasibility and efficacy of HepatiCan™, reinforcing their potential for future clinical application in the treatment of acute liver failure.

One of the most challenging needs, is producing a clinically applicable liver biomass, large enough to fulfil all the complex metabolic functions that the liver performs (Tsiaoussis et al., 2001). The safe limit of liver resection during hepatectomy is ∼30% of the total volume (Guglielmi et al., 2012), which is routinely performed. The lack of efficacy in extended patient survival with the Bioartificial Liver devices tested to date may be partly explained by the insufficient number of cells utilised during trials and lack of means for testing for biomass viability during treatment. Supplementary Table S6 indicates biomass used in different designs. Assuming somewhere between 1 or 2 × 10^11^ cells in the adult human liver, the HepatiCan™ biocartridge biomass constituting a liver mass of 7-8 x 10^10^ cells, is adequate biomass to provide 30% human liver functional requirement for extracorporeal treatment.

The HepatiCan™ device contains HepG2 cell spheroids conditioned and grown in microgravity environment. Cells grown in dynamic conditions within alginate beads form a complex 3-D architecture within cohesive spheroids. Cuboidal cells display excellent cell-to-cell contact, extracellular matrix, desmosomes, microvilli and junctional complexes (Khalil et al., 2001). The spheroids produce extracellular matrix proteins (Selden et al., 2000). This cell-cell contact in liver-like configuration more closely resembles liver tissue than cell lines. Sajiki *et al.*, reported the loss of the polygonal shape in hepatocytes grown in hollow fibre cartridges (Sajiki et al., 2000). Growing HepG2 cells in 3-dimensional gelled alginate culture makes them a suitable candidate cell for Bioartificial Liver therapies.

We discuss the production of a medical grade biocartridge, developed through both computational fluid dynamics and experimental testing of biomass production. The biocartridge is a key component of HepatiCan™, a bioartificial liver (BAL) device designed to support patients with acute liver failure. Technical, clinical and economical challenges must be addressed for its successful clinical translation and commercial viability. Immunogenicity has been minimised by design so that biomass does not enter a patient’s circulation. This is achieved by its extracorporeal use, and treated plasma passes through a 0.6 μm size exclusion safety filter for particle removal and a filter to eliminate endotoxins and allogeneic DNA (Gibbons, 2017). Encapsulation itself acts as a semipermeable barrier, allowing smaller molecules to pass while excluding larger components such as leukocytes. This selective permeability makes alginate a widely used material for encapsulation for cell-based therapies (Xu et al., 2021). No clinically apparent adverse immunologic reactions have been observed in most short-term BAL treatments with systems containing allogeneic or xenogeneic hepatocytes (van de Kerkhove et al., 2004).

HepatiCan™ is designed to provide short-term support—up to 5 days per patient—which aligns well with the needs of acute liver failure management. Cryo-Recovery time is 24–60 h, whilst immediate recovery would be ideal, the typical patient work up would be 1–3 days. The first biocartridge would be used after 24 h and the second replacement biocartridge 48 h later. There is a complex regulatory pathway to follow, however, regulations for a combined ATMP/medicinal product with medical device are becoming better articulated by the regulatory bodies such as MHRA and FDA as more clinical trials are being completed. Good Health Economics data on HepatiCan™ should enable clinical adoption in a cost-effective manner. HepatiCan™ has the potential to be significantly less expensive than orthotopic liver transplantation lifetime costs, approximately $163,000 in United States (van der Hilst et al., 2009), excluding additional expenses like pre-transplant evaluation, immunosuppressive medications, and postoperative care. If HepatiCan™ proves efficacious, it can become a cost-effective bridge-to-transplant or recovery option.

One of the major strengths of the HepatiCan™ therapy is its use of cryopreserved AELS, enabling long term storage and administration on demand. These are critical factors for the treatment of acute liver failure, which develops suddenly and rapidly, requiring immediate treatment. HepatiCan can be delivered in a timely fashion to meet both acute and acute-on-chronic liver insufficiency. In this study, we investigated the suitability of the disposable biocartridge for cell proliferation after encapsulation and during cell recovery after cryopreservation. HepatiCan™ and HepatAssist devices are based on the use of cryopreserved cells, however the latter uses porcine cells (Demetriou et al., 2004). HepatiCan™ therapy is the only approach at clinical scale developed using cryopreserved encapsulated human cell spheroids that could be delivered to patients in a timely fashion.

## 5 Conclusion

This study developed a medical-grade single-use biocartridge for the HepatiCan™ Bioartificial Liver (BAL) device. Computational fluid dynamics were used to theoretically validate the hydrodynamic performance of the new biocartridge. *In vitro* testing demonstrated that the novel single-use biocartridge maintained liver micro-spheroid viability and proliferation. Additionally, function and cell viability were restored after post-thaw culture. In summary, these results demonstrate the biological functionality of the novel, regulatory compliant, HepatiCan™ biocartridge, able to support sufficient biomass, paving the way for subsequent GMP production and clinical trials.

## Data Availability

The raw data supporting the conclusions of this article will be made available by the authors, without undue reservation.

## Abbreviations

AELS, Alginate encapsulated liver spheroids; EAB, Empty alginate beads; FDC, Fluidised dynamic culture; PTC, Post-thaw culture; CFD, Computational fluid dynamics.
