# Supplementary material for: Evaluation of a single-use bioartificial liver (BAL) biocartridge consisting of cryopreservable alginate encapsulated liver cell spheroids as a component of HepatiCan™, a novel bioartificial liver device
Source: Front Bioeng Biotechnol. 2025 Aug 1;13:1572254. doi: 10.3389/fbioe.2025.1572254 (PMC12354383; doi:10.3389/fbioe.2025.1572254)
Supplement: Supplementary file 1 [file Supplementaryfile1.docx]

**Supplementary data**

***
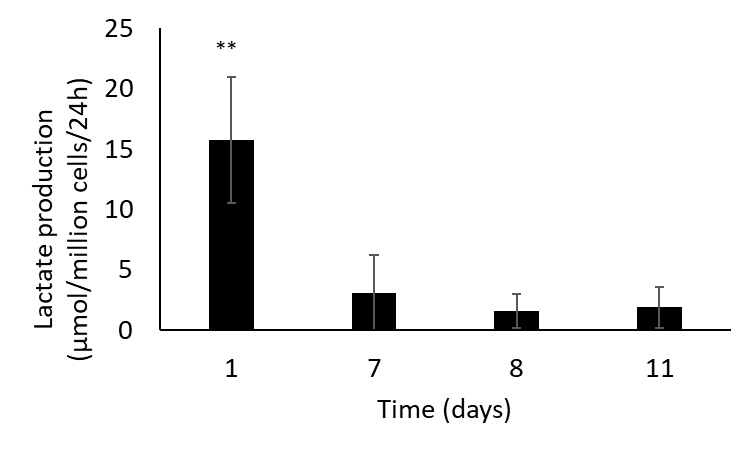
***

***Supplementary Figure S1.*** *Lactate production (µmoles/million cells/24) during cell growth phase. Data shown is n=4, mean ± SD. Statistical analysis assessed by Student’s t-test. *p < 0.05, ***p < 0.001, ****p < 0.0001.*
