# Supplementary material for: Evaluation of a single-use bioartificial liver (BAL) biocartridge consisting of cryopreservable alginate encapsulated liver cell spheroids as a component of HepatiCan™, a novel bioartificial liver device
Source: Front Bioeng Biotechnol. 2025 Aug 1;13:1572254. doi: 10.3389/fbioe.2025.1572254 (PMC12354383; doi:10.3389/fbioe.2025.1572254)
Supplement: Supplementary file 3 [file Table7.docx]

**Supplementary data**

***Supplementary Table 7****. BAL devices with active biomass components reported since 2015.*

| **Device** | **Components** | **Advantages** | **Disadvantages** | **Biomass** |
| --- | --- | --- | --- | --- |
| **Hepatican^TM^ (46,54,56)** | Conditioned human liver cell line (HepG2). Organoids within alginate microspheres in a fluidised bed bioreactor. Extracorporeal secondary Circuit for treatment | Tested to human scale. Cryopreservable for ‘off shelf’ delivery | Preclinical trials only | 7-8 x 10^10^ cells |
| **ELAD (11,13, 40,61)** | Human cell line (HepG2 C3A) in hollow fibre cartridges. Semipermeable membranes housing liver cells in the extra capillary space | Human cell line, no xenogeneic material. Device safety proven in clinical studies. Prospective randomized trial for severe alcoholic hepatitis. | Limited efficacy in acute liver failure. Complex and costly. Clinical trial halted on lack of long-term efficacy. Not cryopreserved | 200-400 grams |
| **SRBAL (7,22,33)** | 3d spheroid culture of porcine hepatocytes. Semipermeable hollow fibre cartridges in a dual-loop extracorporeal circulation system | Improved survival in preclinical studies | Still in preclinical phase. Potential risks of immune responses and porcine endogenous retrovirus transmission | 200-400 grams |
| **hiHep BAL (66)** | Human induced hepatocytes (hiHep). Extracorporeal device | Improved survival in preclinical trials. Potential for scalability. No animal cells. Phase 1 trial in evaluation | Limited early clinical data | 3x10^9^ and 1x10^10^ |
| **HepAssis2*** | Human-derived liver cells (HL2). Semi-automated extracorporeal device | Human-based Cells. HL2 cells maintain normal liver functions. Approved for clinical trials. | Preclinical trial information not available. Regionally approved. Long-term outcomes pending | Not specified |
| **OrganOX eGenesis device*** | Genetically modified pig livers used in an organ perfusion system | Genetically engineered porcine livers. Temporary full-organ function support. Clinical trial approved. | Xenotransplant risks. Early-stage. | Not specified |
| **Stimuliver*** | Pluripotent stem-cell derived hepatocytes. Subcutaneous bioartificial liver implant | Potential for long-term support. Implantable | Preclinical. Unknown immune tolerance | Not specified |

Additional references*: HepAssis2 https://www.togomeditech.com/ and OrganOX eGenesis https://egenesisbio.com/ and Stimuliver https://www.stimuliver.bio
