# Supplementary material for: Evaluation of a single-use bioartificial liver (BAL) biocartridge consisting of cryopreservable alginate encapsulated liver cell spheroids as a component of HepatiCan™, a novel bioartificial liver device
Source: Front Bioeng Biotechnol. 2025 Aug 1;13:1572254. doi: 10.3389/fbioe.2025.1572254 (PMC12354383; doi:10.3389/fbioe.2025.1572254)
Supplement: Supplementary file 4 [file Table2.docx]

**Supplementary data**

***Supplementary Table 2****. Composition of cell culture media used during encapsulation for alginate bead rinsing.*

| Component | Supplier | Catalogue number | Concentration |
| --- | --- | --- | --- |
| DMEM | Sigma-Aldrich | D6429 | - |
| Penicillin / streptomycin | Gibco | 15070063 | 45 U/ml / 45 µg/ml |
| Fungizone | Sigma | A2942 | 1.1 µg/ml |
