# Supplementary material for: Evaluation of a single-use bioartificial liver (BAL) biocartridge consisting of cryopreservable alginate encapsulated liver cell spheroids as a component of HepatiCan™, a novel bioartificial liver device
Source: Front Bioeng Biotechnol. 2025 Aug 1;13:1572254. doi: 10.3389/fbioe.2025.1572254 (PMC12354383; doi:10.3389/fbioe.2025.1572254)
Supplement: Supplementary file 5 [file Table5.docx]

**Supplementary data**

***Supplementary Table 5.*** *Materials and chemicals used for cell counting using the Nucleounter.*

| Component | Supplier | Catalogue number | Concentration |
| --- | --- | --- | --- |
| PBS ^+^Ca ^+^Mg | Gibco | 14040 | - |
| FDA | Sigma | F7378 | 0.0128 mg/mL |
| PI | Biotium | 40016 | 0.0256 mg/mL |
| PBS ^-^Ca ^-^Mg | Gibco | 14190 | - |
| EDTA | Thermo | J15700-A1 | 16mM |
| NaCl | Fisher Scientific | S/3160/65 | 0.15M |
| 21G needle | BD | 304432 | - |
| Reagent A | Chemometec | 910-0003 | - |
| Reagent B | Chemometec | 910-0002 | - |
| Nucleocounter | Chemometec | NC-100 or NC-200 | - |
