# Supplementary material for: Evaluation of a single-use bioartificial liver (BAL) biocartridge consisting of cryopreservable alginate encapsulated liver cell spheroids as a component of HepatiCan™, a novel bioartificial liver device
Source: Front Bioeng Biotechnol. 2025 Aug 1;13:1572254. doi: 10.3389/fbioe.2025.1572254 (PMC12354383; doi:10.3389/fbioe.2025.1572254)
Supplement: Supplementary file 6 [file Table3.docx]

**Supplementary data**

***Supplementary Table 3****. Composition of cell culture media used during fluidised dynamic culture, either in the cell growth phase or post-thaw culture.*

| Component | Supplier | Catalogue number | Concentration |
| --- | --- | --- | --- |
| AlphaMEM | Cytiva | SH3A10135.01 | - |
| FFP | NHSBT | - | 10% |
| Insulin | Novo Nordisk | Actrapid® | 0.27 IU/ml |
| Penicillin / streptomycin | Gibco | 15070063 | 45 U/ml / 45 µg/ml |
| BSA/linoleic acid | Sigma | L9530 | 0.05mg/ml |
| Sodium selenite | Sigma | S5261 | 0.017 µg/ml |
| Hydrocortisone | Sigma | H0888 | 0.364 µg/ml |
| Thyrotropic releasing hormone TRH | Sigma | P1319 | 0.364 µg/ml |
| Fungizone | Sigma | A2942 | 1.1 µg/ml |
| Heparin Sodium | Wockhardt | PL29831/0107 | 80U/mL of plasma |
| D-(+)-glucose | Sigma | G8769 | + 3.57mg/mL |
| 1M CaCl_2_ | Honeywell | 12022 | variable |
