# Supplementary material for: Evaluation of a single-use bioartificial liver (BAL) biocartridge consisting of cryopreservable alginate encapsulated liver cell spheroids as a component of HepatiCan™, a novel bioartificial liver device
Source: Front Bioeng Biotechnol. 2025 Aug 1;13:1572254. doi: 10.3389/fbioe.2025.1572254 (PMC12354383; doi:10.3389/fbioe.2025.1572254)
Supplement: Supplementary file 7 [file Table4.docx]

**Supplementary data**

***Supplementary Table 4.*** *Chemicals used during cryopreservation for freezing and thawing the AELS*

| Component | Supplier | Catalogue number | Concentration |
| --- | --- | --- | --- |
| Me_2_SO (DMSO) | Sigma | 154938 | 12% v/v |
| University of Wisconsin (UW) | Belzer UW® | Cold Storage Solution | 38% v/v |
| Catalase | Sigma | C40 | 500IU/ml |
| Trolox | Aldrich | 238,813 | 1.7mM |
| DMEM | Sigma-Aldrich | D6429 | - |
| D-(+)-glucose | Sigma | G8769 | 1M, 0.5M & 25mM |
