# Supplementary material for: Evaluation of a single-use bioartificial liver (BAL) biocartridge consisting of cryopreservable alginate encapsulated liver cell spheroids as a component of HepatiCan™, a novel bioartificial liver device
Source: Front Bioeng Biotechnol. 2025 Aug 1;13:1572254. doi: 10.3389/fbioe.2025.1572254 (PMC12354383; doi:10.3389/fbioe.2025.1572254)
Supplement: Supplementary file 8 [file Supplementaryfile2.docx]

**Supplementary data**


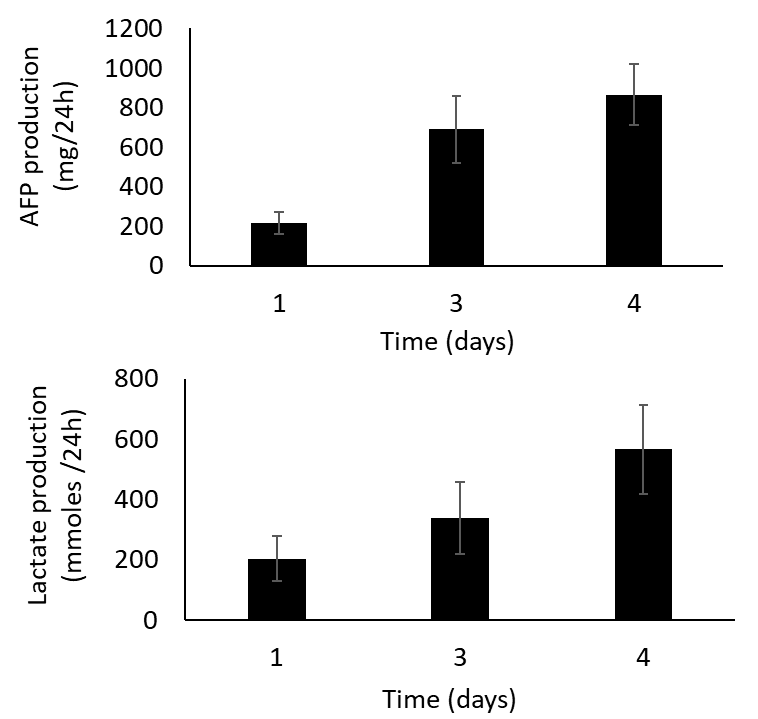


***Supplementary Figure S2.*** *Top: Cell performance during AELS culture: AFP production (mg/24h) during cell recovery after cryopreservation. Data shown is n=4, mean ± SD. Bottom: lactate production (mmoles/24h) during cell recovery after cryopreservation. Data shown is n=4, mean ± SD.*
