# Supplementary material for: Evaluation of a single-use bioartificial liver (BAL) biocartridge consisting of cryopreservable alginate encapsulated liver cell spheroids as a component of HepatiCan™, a novel bioartificial liver device
Source: Front Bioeng Biotechnol. 2025 Aug 1;13:1572254. doi: 10.3389/fbioe.2025.1572254 (PMC12354383; doi:10.3389/fbioe.2025.1572254)
Supplement: Supplementary file 9 [file Table6.docx]

**Supplementary data**

***Supplementary Table 6.*** *Key characteristics of (pre)clinically tested bioartificial liver (BAL) support systems created before 2015, which are not in commercial use, including cell source, bioreactor technology, cryopreservation status, and estimated cell mass.*

| **Device Name** | **Cell Type** | **Technology** | **Cryopreserved Cells** | **Cell Mass (g)** |
| --- | --- | --- | --- | --- |
| **ELAD (11,13,40)** | Human hepatoblastoma cell line (HepG2/C3A) | Hollow fibre cartridges | No | 200–400 g |
| **HepatAssist (9)** | Primary porcine hepatocytes | Hollow fibre bioreactor with microcarriers | Yes | 50–70 g |
| **MELS (53)** | Primary human hepatocytes | Hollow fibre bioreactor | Not specified | Up to 600 g |
| **SRBAL (33)** | Primary porcine hepatocytes cultured as spheroids | Suspension bioreactor with spheroid aggregates | Not specified | Not specified |
| **BLSS (38,48)** | Primary porcine hepatocytes | Hollow fibre bioreactor | Not specified | 70–120 g |
| **AMC-BAL (49,63)** | Primary porcine hepatocytes | Nonwoven polyester matrix with spiral membrane | No | 100 g |
